# The neurobiology of PTSD

**DOI:** 10.1080/20008198.2017.1314165

**Published:** 2017-05-04

**Authors:** Ruth Lanius, Miranda Olff

**Affiliations:** ^a^ Department of Psychiatry, Academic Medical Center, University of Amsterdam & Arq Psychotrauma Expert Group, Diemen, The Netherlands; ^b^ Posttraumatic Stress Disorder (PTSD) Research Unit, University of Western Ontario, London, ONN6A 3K7, Canada

The *European Journal of Psychotraumatology* is proud to announce its first special issue focusing on the neurobiology of posttraumatic stress disorder (PTSD). Since its inception, the journal has published a number of papers on the neural mechanisms underlying PTSD, including review articles on the biological correlates of complex PTSD (Marinova & Maercker, 2015), restoring large scale brain networks in PTSD and related disorders (Lanius, Frewen, Tursich, Jetly, & McKinnon, 2015), and pharmacological treatments and their neurobiological underpinnings (Kelmendi et al., 2016). In addition, methodological considerations when investigating epigenetic consequences of early life adversity have been outlined (Fiori & Turecki, 2016). Finally, an article calling for clinical, treatment, and neuroscience research in the area of trauma-related dissociation and altered states of consciousness was published as part of a special issue focusing on setting the research agenda in PTSD and its underlying neurobiology (Lanius, 2015). Few open access journals publish neurobiological research, while we do feel very strongly about also making this type of research publicly available without any barriers of paid access.

The current special issue includes both review articles and original research papers. The first review article stresses the importance of being aware of cultural differences in self-representation and their effects on the neural correlates of PTSD (Liddell & Jobson, 2016). The authors outline five important affective and cognitive functions critical to PTSD, including fear dysregulation, attentional biases to threat, emotion and autobiographical memory, self-referential processing, and attachment and interpersonal functioning and describe how these functions and their neural correlates can be influenced by cultural differences in self-representation. The review concludes with a conceptual model emphasizing how the five affective and cognitive functions described above are crucial to how culture may inform the neural correlates underlying trauma-related disorders (Liddell & Jobson, 2016). The second review summarizes the neurobiological and neuroendocrine correlates underlying individuals with PTSD who continue to face ongoing threat and stresses the need to distinguish neuroendocrine findings in individuals facing ongoing threat versus those who do not (Fragkaki, Thomaes, & Sijbrandij, 2016).

The first original research article examines potential mechanisms underlying eye movement desensitization and reprocessing (EMDR) by studying brain activation patterns during traumatic memory recall with and without eye movements (Thomaes, Engelhard, Sijbrandij, Cath, & Van den Heuvel, 2016). Results suggest that performing eye movements during traumatic memory recall may be associated with decreased activation and connectivity in brain regions involved in emotion processing, including the rostral anterior cingulate cortex and the amygdala. The second research article presents a neuroimaging investigation examining the relationship between early life adversity and theory of mind demonstrates increased activation of the left inferior frontal gyrus in individuals with a history of sexual abuse in the context of emotional abuse and neglect. The authors propose that this may interfere with interpersonal functioning in individuals with early life adversity in complex social situations (van Schie et al., 2017). The third research article compares self-referential processing to other-referential processing in PTSD predominantly related to childhood and interpersonal trauma (Frewen, Thornley, Rabellino, & Lanius, 2017). Individuals with PTSD often experience pervasive negative emotional states, including anger, guilt, and shame (Frewen, Schmittmann, Bringmann, & Borsboom, 2013; Stotz, Elbert, Müller, & Schauer, 2015; Taylor, 2015). Results demonstrated that individuals with PTSD endorsed *more* negative words and less positive words when describing themselves and others, respectively. Neuroimaging findings demonstrated altered connectivity of key brain regions involved in self-referential processing, including the default mode network, during self- and other-referential processing. These findings have implications for treatment focusing on the processing of self and others in PTSD.

In summary, we have received a range of articles focusing on key emerging neurobiological and clinical concepts in PTSD. We look forward to receiving more papers focusing on the neurobiology underlying trauma-related disorders in order to make this area an integral part of the *European Journal of Psychotraumatology*.
